# Evaluation of the anticancer potential of six herbs against a hepatoma cell line

**DOI:** 10.1186/1749-8546-7-15

**Published:** 2012-06-10

**Authors:** Natthida Weerapreeyakul, Apiyada Nonpunya, Sahapat Barusrux, Thaweesak Thitimetharoch, Bungorn Sripanidkulchai

**Affiliations:** 1 Faculty of Pharmaceutical Sciences, Khon Kaen University, Khon Kaen, 40002, Thailand; 2 Center for Research and Development of Herbal Health Products (CRD-HHP), Faculty of Pharmaceutical Sciences, Khon Kaen University, 123 Mittrapap Road, Muang District, Khon Kaen, 40002, Thailand; 3 Graduate School, Faculty of Pharmaceutical Sciences, Khon Kaen University, Khon Kaen, 40002, Thailand; 4 Center for Research and Development of Medical Diagnostic Laboratories (CMDL), Khon Kaen University, Khon Kaen, 40002, Thailand; 5 Faculty of Associated Medical Sciences, Khon Kaen University, Khon Kaen, 40002, Thailand

## Abstract

**Background:**

Six herbs in the Plant Genetics Conservation Project that have been used as complementary medicines were chosen on the basis of their medicinal value, namely *Terminalia mucronata*, *Diospyros winitii*, *Bridelia insulana*, *Artabotrys harmandii*, *Terminallia triptera*, and *Croton oblongifolius*. This study aims to evaluate the potential anticancer activity of 50% ethanol-water extracts of these six herbs.

**Methods:**

Fifty percent ethanol-water crude extracts of the six herbs were prepared. The cytotoxicity of the herbal extracts relative to that of melphalan was evaluated using a hepatoma cell line (HepG2), and examined by neutral red assays and apoptosis induction by gel electrophoresis and flow cytometry after 24 h.

**Results:**

A significant difference was found between the cytotoxicity of the 50% ethanol-water crude extracts and melphalan (*P* = 0.000). The 50% ethanol-water crude extracts of all six herbs exhibited cytotoxicity against HepG2 cells, with IC_50_ values ranging from 100 to 500 μg/mL. The extract of *T. triptera* showed the highest cytotoxicity with an IC_50_ of 148.7 ± 12.3 μg/mL, while melphalan had an IC_50_ of 39.79 ± 7.62 μg/mL. The 50% ethanol-water crude extracts of *D. winitii* and *T. triptera*, but not *A. harmandii*, produced a DNA ladder. The 50% ethanol-water crude extracts of *D. winitii*, *T. triptera*, and *A. harmandii* induced apoptosis detected by flow cytometry.

**Conclusion:**

The 50% ethanol-water crude extracts of *D. winitii*, *T. triptera*, and *A. harmandii* showed anticancer activity *in vitro*.

## Background

The Plant Genetics Conservation Project, under the patronage of Her Royal Highness Princess Maha Chakri Sirindhorn, aims to evaluate the biochemical activities and properties of potential medicinal plants. Previously, cytotoxic and apoptotic effects of medicinal plants found in Chaiyaphum province have been discovered [1]. Therefore, subsequent studies were extended for herbs in other Plant Genetics Conservation Project areas, including *Terminalia triptera* Stapf and *Terminalia mucronata* Craib & Hutch in Combretaceae, *Croton oblongifolius* Roxb. and *Bridelia ovata* Decne in Euphorbiaceae, *Diospyros winitii* Fletcher in Ebenaceae, and *Artabotrys harmandii* Finet & Gagnep. in Annonaceae. Except for *T. mucronata* and *A. harmandii*, the other four herbs are all found in China [2,3].

The abovementioned herbs are commonly used in Asia [2,3]. For example, the bark of *T. triptera* is used with betel nuts as a remedy for aphthous ulcer [3]. Meanwhile, for *C. oblongifolius*, its leaves are used as a tonic for indigestion, flatulence, and bruises, its flowers for the treatment of flat worms, its fruit for dysmenorrhea, its seeds as a purgative, its roots for dysentery, and its bark for dyspepsia, chronic hepatomegaly, and fever [3]. The medicinal use of *D. winitii* is unclear*.* The longstanding medicinal use of these herbs necessitates research into their clinical safety and rigorous biological activity testing. Since cancer is a leading cause of death worldwide [4], evaluation of the anticancer activity of these herbs is the first priority. It was previously reported that labdane diterpenoids extracted from the stem bark of *C. oblongifolius* exert moderate cytotoxicity toward cancer cell lines [5].

In this study, determination of anticancer activity was based on the cytotoxicity of 50% ethanol-water crude extracts toward a human hepatocarcinoma cell line, HepG2. Cytotoxicity and selectivity were determined for the safety of the 50% ethanol-water crude extracts using a normal African green monkey kidney epithelial cell line, Vero, relative to the HepG2 cell line. Apoptotic cell death is a protective mechanism that destroys misprogrammed or nonfunctional cells and is used as a clinical endpoint for effective anticancer treatment [6,7]. The apoptosis-inducing effects were determined in HepG2 cells by examination of DNA fragmentation, which is observed as DNA laddering by agarose gel electrophoresis. The apoptosis-inducing effects of the plant extracts were further confirmed using Annexin V-FITC staining and detection by flow cytometry.

In this study, we aims to investigate the anticancer activity of six herbs based on their cytotoxicity and apoptosis induction.

## Methods

### Chemicals and reagents

Dulbecco’s modified Eagle’s medium (DMEM), fetal bovine serum (FBS), penicillin, and streptomycin were purchased from GIBCO® (Invitrogen, USA). Dimethylsulfoxide (DMSO) and ethidium bromide were purchased from Sigma-Aldrich (USA). Sodium bicarbonate, neutral red (NR), and melphalan in powder form were purchased from Sigma-Aldrich (Germany). Isopropyl alcohol (biotechnology grade) was purchased from Bio Basic Inc. (USA). Methanol (analytical grade) was purchased from BDH (England). A FlexiGene DNA Kit was obtained from Qiagen (Germany). Agarose (molecular grade) was purchased from Bio-Rad (USA), and an 100 bp + 1.5 Kb DNA ladder with stain was purchased from SibEnzyme (Russia). All other reagents used in this study were purchased from Sigma Chemicals Co. (USA). An Annexin V-FITC Apoptosis Detection Kit was purchased from Bender MedSystems GmbH (Austria).

## Herb materials

Twigs of the six herbs were collected and authenticated by Assistant Professor Thaweesak Thitimetharoch based on the taxonomy [TT, OC & SK-number]. The vouchers were deposited at the Herbal Herbarium, Faculty of Pharmaceutical Sciences, Khon Kaen University, Khon Kaen Province, Thailand. The details of the herbs and voucher numbers are summarized in Table 1.

**Table 1:** Cytotoxicity of the 50% ethanol-water crude extracts based on the neutral red assay, and the scientific name, family, part used and % yield

**Scientific name (Voucher specimen no.)**	**Family**	**% yield per gram dry weight**	**Cytotoxicity (IC_50_ value, μg/mL)**	
*T. triptera*	Combretaceae	6.14	136.4 ± 44.5	148.7 ± 12.3
[TT, OC & SK No 1214, 1224]				
*C. oblongifolius*	Euphorbiaceae	6.18	238.9 ± 21.9	378.4 ± 18.7
[TT, OC & SK No 1215, 1256]				
*D. winitii*	Ebenaceae	4.13	> 500	204.7 ± 33.5
[TT, OC & SK No 1245]				
*B. ovata*	Euphorbiaceae	5.68	> 500	183.9 ± 7.1
[TT, OC & SK No 1253]				
*A. harmandii*	Annonaceae	3.28	309.7 ± 58.2	163.5 ± 11.7
[TT, OC & SK No 1264]				
*T. mucronata*	Combretaceae	3.93	129.4 ± 18.4	152.0 ± 33.2
[TT, OC & SK No 1269]				
Melphalan			59.9 ± 3.2	37.7 ± 9.8

### Herb extractions

Fifty percent ethanol-water crude extracts were prepared from the dried stems of each herb, which were cut and macerated with 50% ethanol-water at a ratio of 1 kg/6 L for 7 days with occasional shaking. The solvent was filtered, distilled *in vacuo* using a rotary evaporator (Rotavapor R-200; Büchi Labortechnik AG, Switzerland) below 40°C, and freeze-dried to obtain the 50% ethanol-water crude extracts. The 50% ethanol-water extracts were freshly prepared as stock solutions in DMSO.

### Gas chromatography/mass spectrometry (GC-MS) analysis

A gas chromatography system (Model 6890 N; Agilent Technologies, China) coupled with a mass selective detector (Model 5973 N; Agilent Technologies, USA) and a GC autosampler (HP 7683 series) were employed for all analyses. Samples were separated on a 30.0-m long, 250-μm diameter, 0.25-μm film thickness, 122–5532 DB-5 ms capillary column from Agilent Technologies (J&W Scientific, USA). The column initially flowed at 80°C for 6 min with a rate of 2 mL/min and an average velocity of 52 cm/s. The temperature was then raised to 280°C (at a rate of 5°C/min) and maintained for 24 min. The total runtime was 70 min. Ultrapure helium with an inline Alltech oxygen trap was used as the carrier gas (19.34 psi), with the purge flow set at 20.0 mL/min, the purge time set at 0.75 min, and a total flow of 24.3 mL/min. The injector temperature was maintained at 250°C, and the injection volume at 2.0 μL in the splitless mode. The interface temperature was held at 280°C. Mass spectra were scanned from *m*/*z* 50.0 to *m*/*z* 500.0 at a rate of 1.5 scans/s with a threshold of 150. The electron impact ionization energy was 70 eV. The chemical components of the crude extracts were identified from the chromatograms and mass spectra using the Wiley 7 N.l database (Agilent Technologies).

### Cell culture

The HepG2 and Vero cell lines were maintained at the Center for Research and Development of the Medical Diagnostic Laboratories, Khon Kaen University. DMEM was supplemented with 10% FBS, 100 U/mL penicillin, and 100 μg/mL streptomycin. The cells were cultured at 37°C under a humidified atmosphere containing 5% CO_2_.

### Cytotoxicity based on NR assays

The stock solutions of the herb extracts in DMSO were diluted with DMEM to the desired concentrations (10–500 μg/mL). The maximum concentration of the 50% ethanol-water extracts in each test was 500 μg/mL, such that the final concentration of DMSO would not exceed 1% (v/v) and the cytotoxicity of DMSO would be less than 10%. The HepG2 hepatoma cell line and normal Vero cell line were used as the cell models. The cytotoxicity evaluation was performed using NR assays according to Fotakis and Timbrell [8], with some modifications. Melphalan was used as a standard anticancer drug for comparisons with the crude extracts.

The cells were seeded in 96-well plates at a density of 3 × 10^5^ cells/mL and treated with different concentrations of the 50% ethanol-water crude extracts or melphalan for 24 h. The cells were then washed and the supernatants were discarded. The NR was dissolved in 100 μL of 0.33% HCl. The NR solution was added to each well, yielding a final concentration of 50 μg/mL, and incubated at 37°C for 1 h. The absorbance of the NR dye was detected using a dual-wavelength UV spectrometer (Anthos 2010; Biochrom, UK) at 520 nm with a reference wavelength of 650 nm. The percent cytotoxicity compared with untreated cells was determined and calculated as the %-cytotoxicity. A plot of %-cytotoxicity *vs.* test compound concentration was used to calculate the IC_50_. The selectivity index (SI) was calculated from the IC_50_ of the 50% ethanol-water crude extracts in normal cells *vs.* cancer cells to indicate the cytotoxic selectivity (*i.e.*, safety) of the 50% ethanol-water crude extracts against cancer cells *vs.* normal cells.

### Apoptosis induction assays

#### *DNA fragmentation detection assay*

DNA fragmentation was used as a proxy for apoptosis induction. After treatment of cancer cells with 500 μg/mL of the 50% ethanol-water extracts and 80 μg/mL of melphalan for 24 h, the cells were collected and washed with medium. The cell suspensions were transferred to microcentrifuge tubes (1.5 mL) and centrifuged at 500 × *g* (PMI-Labortechnik GmbH, Germany) for 5 min, followed by collection of the cell pellets. The DNA in the cell pellets was extracted using a FlexiGene DNA Kit according to the web-published FlexiGene® DNA Handbook. Aliquots (2 μg) of the DNA were analyzed by electrophoresis in 1.8% agarose gels containing 0.1% ethidium bromide. After the electrophoresis, the DNA fragments were analyzed using a UV-illuminated camera (Syngene, UK).

#### *Annexin V-FITC staining for apoptosis detection by flow cytometry*

An Annexin V-FITC and propidium iodide (PI) double staining method was used according to Moosavi and Yazdanparast [9], with some modifications. The cells were seeded in a 24-well plate at a density of 5 × 10^5^ cells/mL, supplemented with DMEM containing 10% FBS, and incubated in a 5% CO_2_ incubator at 37°C for 24 h. The test compounds were added to the wells to give final concentrations of 500 μg/mL for the respective 50% ethanol-water extracts and 80 μg/mL for melphalan. The exposure time between the cells and the test compounds was 24 h. Subsequently, the cells were washed with 1 × phosphate-buffered saline (PBS), trypsinized, and transferred to microcentrifuge tubes (1.5 mL). The cells were centrifuged at 500 × *g*, and the cell pellets were resuspended with 195 μL of binding buffer (10 mM HEPES/NaOH pH 7.4, 140 mM NaCl, 2.5 mM CaCl_2_). The cells were then stained with 5 μL of Annexin V-FITC in the dark at room temperature for 5 min and centrifuged at 500 × *g* to collect the cell pellets. Next, the cell pellets were resuspended in 190 μL of binding buffer and stained with 10 μL of PI for 10 min under the same conditions. Finally, the fluorescence intensity was determined using a flow cytometer (FACSCanto II; BD Biosciences, USA), and the percentages of cells in different populations were quantified using a quadrant marker.

### Statistical analysis

The data were represented as means ± standard deviation (SD) (n = 3). The statistical significance of differences in multiple-group comparisons were evaluated by a one-way analysis of variance followed by Tukey’s Honestly Significant Difference test using SPSS version 11.5 (SPSS Inc., USA). A two-tailed Student’s *t*-test was also performed. Values of *P* < 0.05 were considered statistically significant within a 95% confidence interval.

## Results and discussion

The 50% ethanol-water crude extracts of the six herbs showed moderate cytotoxicity toward HepG2 and Vero cells, except for the lack of activity of the *D. winitii* and *B. ovata* extracts toward Vero cells (IC_50_ > 500μg/mL).

The 50% ethanol-water crude extracts of the six herbs exhibited different degrees of cytotoxicity toward HepG2 cells, with IC_50_ values ranging from 100 to 500 μg/mL, and showed significant differences from untreated cells (*P* = 0.000). The *T. triptera* extract showed high cytotoxicity toward HepG2 cells (IC_50_ = 148.7 ± 12.3 μg/mL). A significant difference was found between the cytotoxicity of melphalan and the 50% ethanol-water crude extracts (*P* = 0.000). All of the 50% ethanol-water crude extracts showed significantly lower cytotoxicity than melphalan (IC_50_ = 37.7 ± 9.8 μg/mL) (*P* = 0.000).

*T. triptera* and *T. mucronata*, which are both in the genus Terminalia of the family Combretaceae, were the most cytotoxic herbs toward HepG2 cells. In HepG2 cells, no significant differences in cytotoxicity were observed between *T. triptera* and *T. mucronata* (*P* = 1.000), *T. triptera* and *A. harmandii* (*P* = 0.862), *D. winitii* and *B. ovate* (*P* = 1.000), *D. winitii* and *A. harmandii* (*P* = 0.419), *B. ovata* and *A. harmandii* (*P* = 0.385), and *T. mucronata* and *A. harmandii* (*P* = 0.862). For Vero cells, no significant differences in cytotoxicity were observed between *D. winitii* and *B. ovata* (*P* = 1.000) and *T. triptera* and *T. mucronata* (*P* = 0.999).

The cytotoxicity and SI values of the plant extracts for HepG2 cells *vs.* Vero cells are summarized in Table 2. The extracts showing significant differences in cytotoxicity (Student’s *t*-test) toward HepG2 cells compared with normal Vero cells similar to that of melphalan (*P* = 0.000) were *C. oblongifolius* (*P* = 0.002), *D. winitii*, (*P* = 0.000), *B. ovata* (*P* = 0.000), and *A. harmandii* (*P* = 0.005). None of the extracts had an SI value of > 3 for classification as highly selective.

**Table 2:** **Classification of the cytotoxicity and selectivity index (SI) of the 50% ethanol-water crude extracts in HepG2 cells *vs. *Vero cells**

**Cytotoxicity**	**SI Value**	**HepG2 *vs. *Vero**
Very strong cytotoxicity	SI ≥ 3	-
(IC_50_ < 10 μg/mL)		
Strong cytotoxicity	SI ≥ 3	-
(IC_50_ 10 - 100 μg/mL)	SI < 3	-
Moderate cytotoxicity	SI ≥ 3	-
(IC_50_ 100 - 500 μg/mL)	SI < 3	
		*B. ovata* (> 2.72)
		*D. winitii* (> 2.44)
		*A. harmandii* (1.89)
		*T. triptera* (0.92)
		*T. mucronata* (0.85)
		*C. oblongifolius* (0.63)

**Figure 1:**
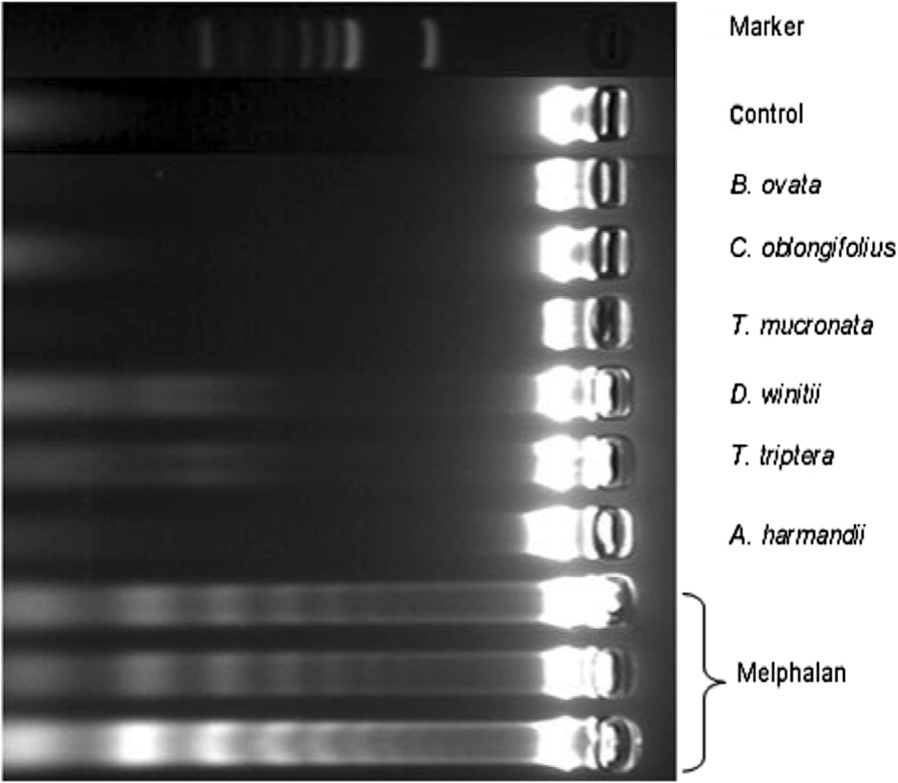
**DNA laddering in apoptotic HepG2 cells.** DNA laddering was visualized in HepG2 cells after treatment with 80 μg/mL of melphalan and 500 μg/mL of 50% ethanol-water crude extracts for 24 h.

The 50% ethanol-water extracts had only moderate anticancer activity, as evaluated by apoptosis induction. The distinct DNA ladder in HepG2 cells showed the characteristics of late-stage apoptosis after treatment with melphalan for 24 h (Figure 1). The extracts of *D. winitii* and *T. triptera* showed subtle DNA ladders, while the other extracts did not induce the late-stage of apoptosis in HepG2 cells

However, the DNA ladder assay has lower sensitivity for apoptosis detection than flow cytometry [10], because DNA ladder formation can only be clearly observed when the extent of oligonucleosomal cleavage is remarkable. Moreover, necrotic cells can also generate DNA fragments [11]. Therefore, the apoptosis findings obtained by Annexin V/PI staining, as detected by flow cytometry, may provide more details.

**Figure 2:**
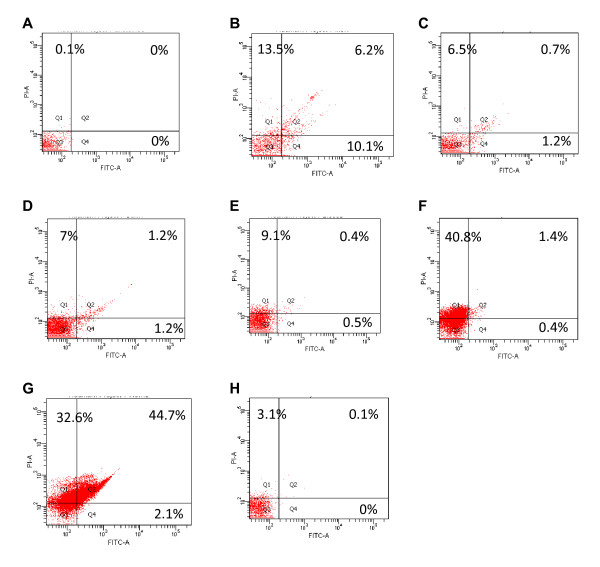
**Percent cell populations at different death modes and percent viable cells detected by Annexin V-FITC staining and flow cytometry after treatment of HepG2 cells with 80 μg/mL of melphalan and 500 μg/mL of 50% ethanol-water crude extracts for 24 h. A**: Untreated cells; **B**: melphalan-treated cells; **C**: *Diospyros winitii*-treated cells; **D**: *Terminalia triptera*-treated cells; **E**: *Terminalia mucronata*-treated cells; **F**: *Croton oblongifolius*-treated cells; **G**: *Artabotrys harmandii*-treated cells; **H**: *Bridelia ovata*-treated cells.

Percent cell populations at different death modes detected by Annexin V-FITC staining and flow cytometry after HepG2 cells treated with melphalan and 50% ethanol-water extracts for 24 h were shown in Figure 2. Results show that four extracts induced apoptosis *vs.* untreated HepG2 cells at the late stage, with the most effective being *A. harmandii*, *T. triptera*, *D. winitii*, and *C. oblongifolius*. Melphalan induced 10.1% early-stage apoptosis and 6.2% late-stage apoptosis, and also caused 13.5% necrosis. Melphalan induced a significantly different %-apoptosis than the plant extracts (*P* = 0.000). Moreover, significant differences in the mean %-apoptosis were observed between *D. winitii* and *A. harmandii* (*P* = 0.000), T *triptera* and *A. harmandii* (*P* = 0.000), *T. mucronata* and *A. harmandii* (*P* = 0.000), *C. oblongifolius* and *A. harmandii* (*P* = 0.000), and *A. harmandii* and *B. ovata* (*P* = 0.000). Among the six herbs, the *A. harmandii* extract induced the highest degree of apoptosis by causing 2.1% early-stage apoptosis and 44.7% late-stage apoptosis with 32.6% high necrosis in HepG2 cells.

*B. ovata*, *D. winitii*, and *A. harmandii* were potentially cytotoxic with relatively high selectivity for HepG2 cells. Only *A. harmandii* showed high apoptosis in HepG2 cells (especially late-stage apoptosis) and a relatively high necrosis effect. *B. ovata* and *D. winitii* induced relatively higher necrosis in HepG2 cells than apoptosis, based on Annexin V/PI staining. These data suggested that the cell death among HepG2 cells induced by these two herbs was mostly necrosis. Since necrosis is an irreversible inflammatory form of cell death, it has some implications for cancer therapy [12]. Thus, *B. ovata* and *D. winitii* may be of interest for further investigations into their anticancer activity *via* necrosis as the mode of death.

Since 50% ethanol-water crude extracts were used in the present study, standardization of the three potential crude extracts for future quality control experiments was necessary. We then determined whether the phytochemicals in the crude extracts were associated with the apoptosis effects. GC-MS analyses of the *D. winitii*, *T. triptera*, and *A. harmandii* extracts were conducted. The GC peaks obtained were used for further chemical constituent identification using the MS database. The GC-MS analyses of the three crude extracts are shown in Additional files 1, 2, and 3 and the main chemical constituents are presented in Table 3.

**Table 3:** GC-MS profiles of the three potential plant 50% ethanol-water crude extracts

**Plants**	**Retention time (min)**	**% of total area**	**Mass spectra**	**Assigned compounds**
T *triptera*	11.94	7.126	51, 65, 91, 119, 120	4-Vinylphenol
	12.29	6.335	53, 63, 69, 81, 97, 109, 126	5-Hydroxymethyl-2-furancarboxaldehyde
	29.52	3.220	60, 73, 83, 97, 115, 129, 143, 157, 171, 185, 199, 213, 227, 256	n-Hexadecanoic acid
	36.34	5.579	57, 71, 107, 135, 320	3-Bis(methylthio)methylene-2-methyl-4-(methoxyphenyl)
*D. winitii*	14.45	1.784	51, 77, 107, 135, 150	2-Methoxy-4-vinylphenol
	15.44	2.605	65, 96, 111, 139, 154	Phenol,2,6-dimethoxy-
	21.92	2.464	111, 126, 141, 169, 184	Acetaldehyde,(3-chloro-5,5-dimethyl-2-cyclohexane-1-ylidene)
	24.85	8.367	77, 91, 124, 137, 180	1,7-Dimethyl-4,4a,5,6-tetrahydropyrido-1H[1,2-b]pyridazin-2)3H)one
	29.53	8.318	60, 73, 83, 97, 115, 129, 143, 157, 171, 185, 199, 213, 227, 256	n-Hexadecanoic acid
	36.35	5.656	57, 107, 135, 152, 320	4-Trimethylsilyl-3-(1-phenylthiobutyl)pent-3-en-2-one
*A. harmandii*	14.43	2.260	51, 77, 107, 135, 150	2-Methoxy-4-vinylphenol
	24.88	10.966	77, 91, 124, 137, 180	2-(2-Oxoethyl)-cis-bicyclo[3.3.0]octane-3,7dione
	29.64	8.398	60, 73, 83, 97, 115, 129, 143, 157, 171, 185, 199, 213, 227, 256	Hexadecanoic acid
	39.10	27.801	57, 74, 98, 112, 134, 239, 257, 299	Hexadecanoic acid, 2-hydroxy-1-(hydroxymethyl)ethyl ester)
	42.17	18.451	43, 57, 74, 98, 112, 134, 154, 267, 284, 298, 327, 341, 358	Glyceryl monosterate

The GC chromatograms of the *T. triptera*, D*. winitii*, and *A. harmandii* extracts differed significantly. A GC peak of the retention time ranging from 23 to 24 min was found in the GC chromatograms of the three crude extracts, indicating that they may contain similar constituents. This peak region could not be identified by MS because the peaks were merged. The MS profiles of all of the crude extracts revealed that the crude extracts of the three plants had two identical compounds, namely vinylphenol and hexadecanoic acid, which are the most common phenolic and fatty acids in plants. Based on the identification of the extant phytochemicals in the 50% ethanol-water crude extracts using the MS database, the 50% ethanol-water crude extracts possessed the same GC retention time. However, the whole MS spectra of the individual 50% ethanol-water crude extracts appeared different, indicating the presence of different compounds.

This study is the first report of cell-based assays for the anticancer effects of selected plants. *A. harmandii* was considered to have the most potential for further study regarding its apoptosis-inducing mechanism, based on the findings of the flow cytometry analyses. Its bioactive constituents and anticancer activity toward other cancer cell types should be determined.

## Conclusions

The 50% ethanol-water crude extracts of *D. winitii*, *T. triptera*, and *A. harmandii* showed anticancer activity *in vitro*.

## Abbreviations

DMEM: Dulbecco’s modified Eagle’s medium; DMSO: dimethylsulfoxide; FBS: fetal bovine serum; GC-MS: gas chromatography/mass spectrometry; NR: neutral red; PBS: phosphate-buffered saline; PI: propidium iodide; SI: selectivity index; UV: ultraviolet.

## Competing interests

The authors declare that they have no competing interests.

## Authors' contributions

NW designed the study, supervised the experiments, and wrote and revised the manuscript. AN performed the experiments. TT performed the plant authentication. BS provided the plant extracts. All the authors read and approved the final version of the manuscript.

## Supplementary Material

Additional file 1 **(A) GC-MS chromatogram 10 mg/mL *T. triptera *crude extract in DMSO.** (B-E) Mass spectra of the crude extract with a respective retention time of 11.94, 12.29, 29.52, and 36.34 min. Click here for file

Additional file 2 **(A) GC-MS chromatogram of 10 mg/mL *D. winitii * crude extract in DMSO.** (B-G) Mass spectra of the crude extract with a respective retention time of 14.45, 15.44, 21.92, 24.85, 29.53 and 36.35 min. Click here for file

Additional file 3 **(A) GC-MS chromatogram of 10 mg/mL *A. harmandii *crude extract in DMSO.** (B-F) Mass spectra of the crude extract with a respective retention time of 14.43, 24.88, 29.6, 39.10 and 42.17 min. Click here for file
