# Supplementary material for: Evaluation of the anticancer potential of six herbs against a hepatoma cell line
Source: Chin Med. 2012 Jun 10;7:15. doi: 10.1186/1749-8546-7-15 (PMC3502167; doi:10.1186/1749-8546-7-15)
Supplement: Additional file 1 — (A) GC-MS chromatogram 10 mg/mL T. triptera crude extract in DMSO. (B-E) Mass spectra of the crude extract with a respective retention time of 11.94, 12.29, 29.52, and 36.34 min. [file 1749-8546-7-15-S1.doc]

(A)


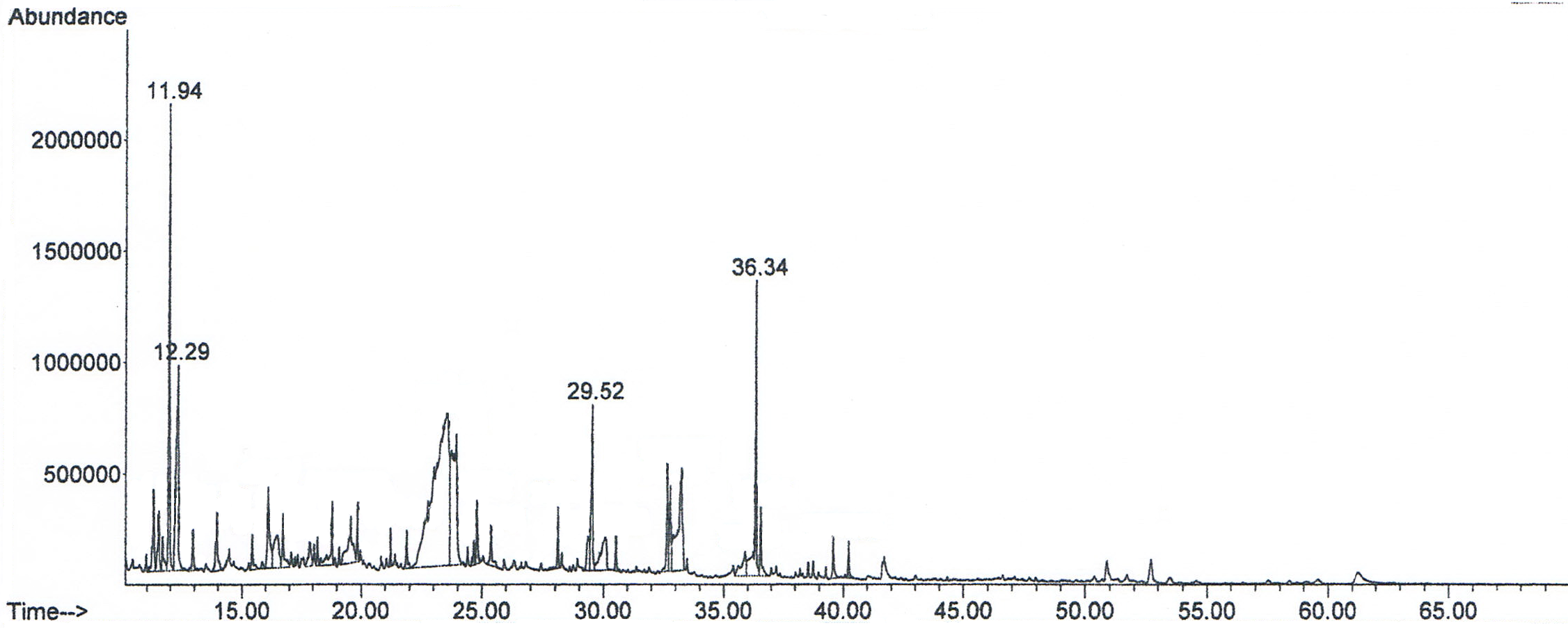


(B)


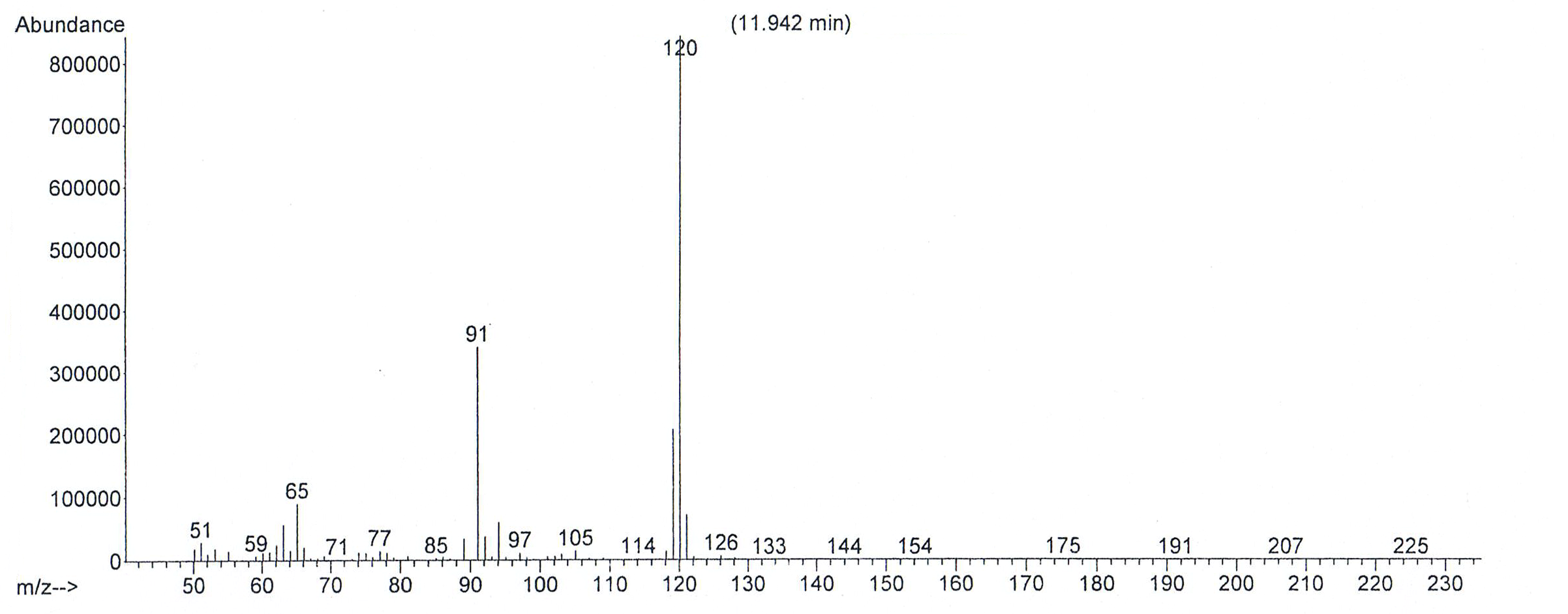


(C)


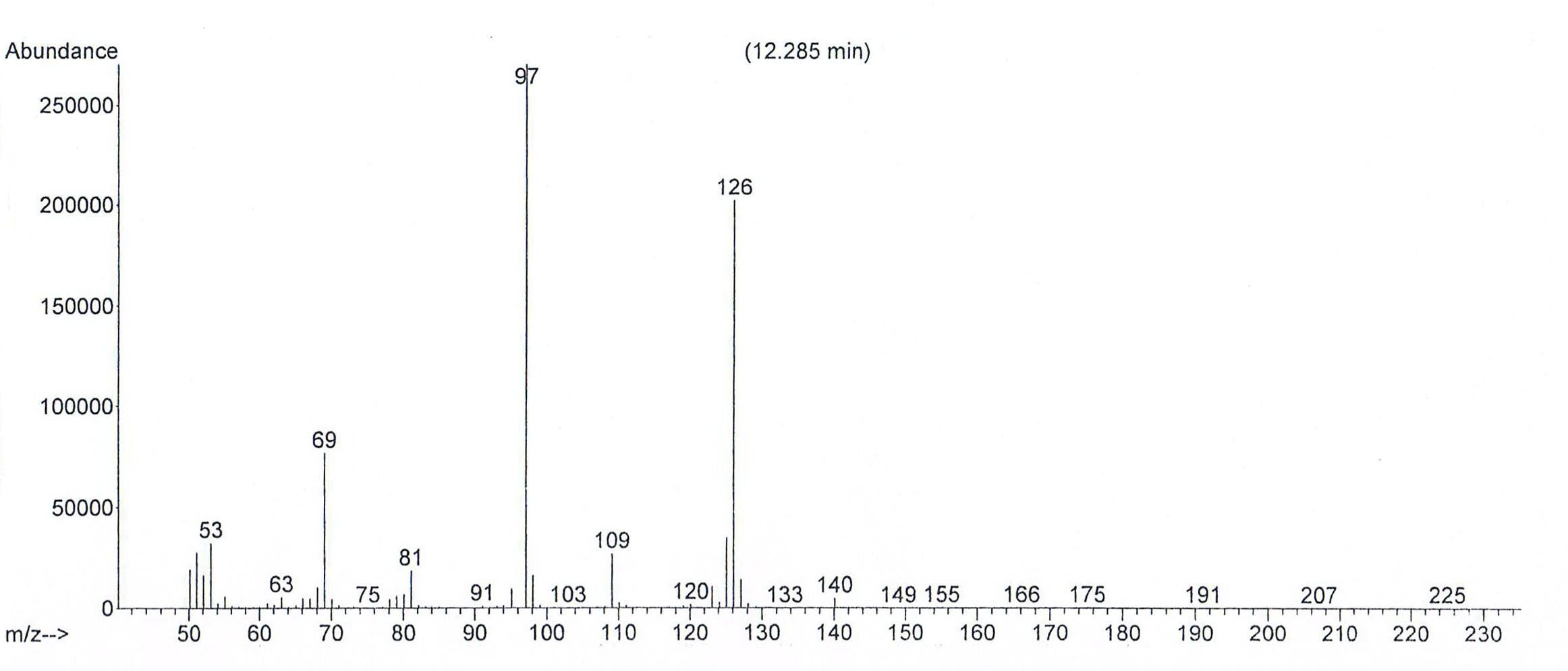


(D)


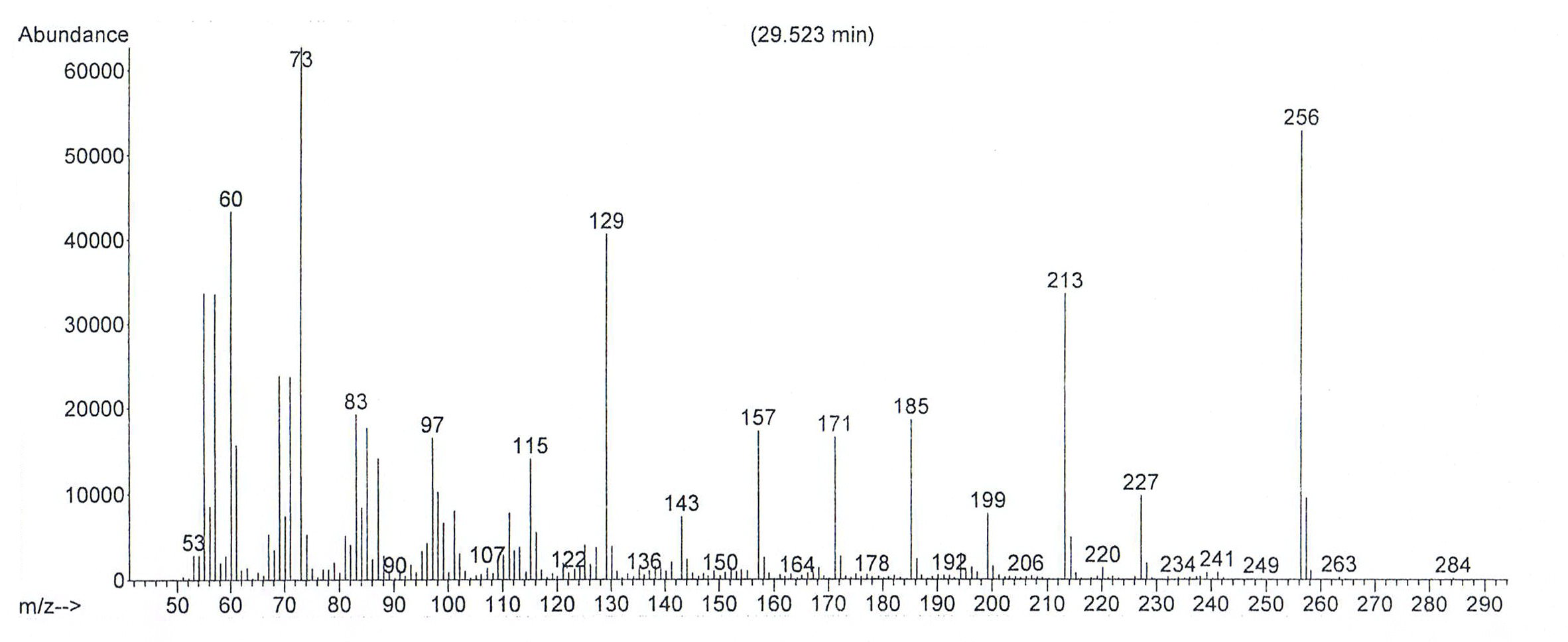


(E)


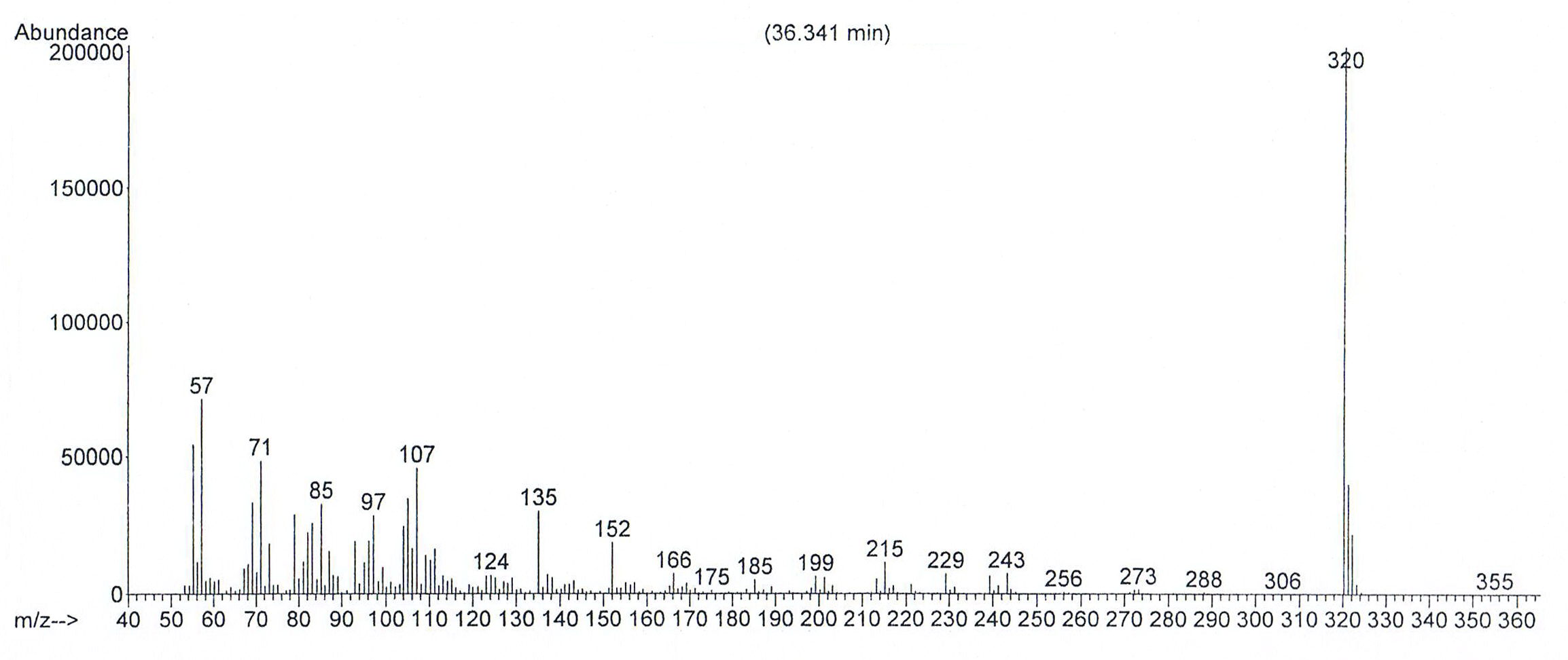


**Additional file 1**: (A) GC-MS chromatogram 10 mg/mL *T. triptera* crude extract in DMSO. (B-E) Mass spectra of the crude extract with a respective retention time of 11.94, 12.29, 29.52, and 36.34 min.
