# Supplementary material for: Evaluation of the anticancer potential of six herbs against a hepatoma cell line
Source: Chin Med. 2012 Jun 10;7:15. doi: 10.1186/1749-8546-7-15 (PMC3502167; doi:10.1186/1749-8546-7-15)
Supplement: Additional file 2 — (A) GC-MS chromatogram of 10 mg/mL D. winitii crude extract in DMSO. (B-G) Mass spectra of the crude extract with a respective retention time of 14.45, 15.44, 21.92, 24.85, 29.53 and 36.35 min. [file 1749-8546-7-15-S2.doc]

(A)


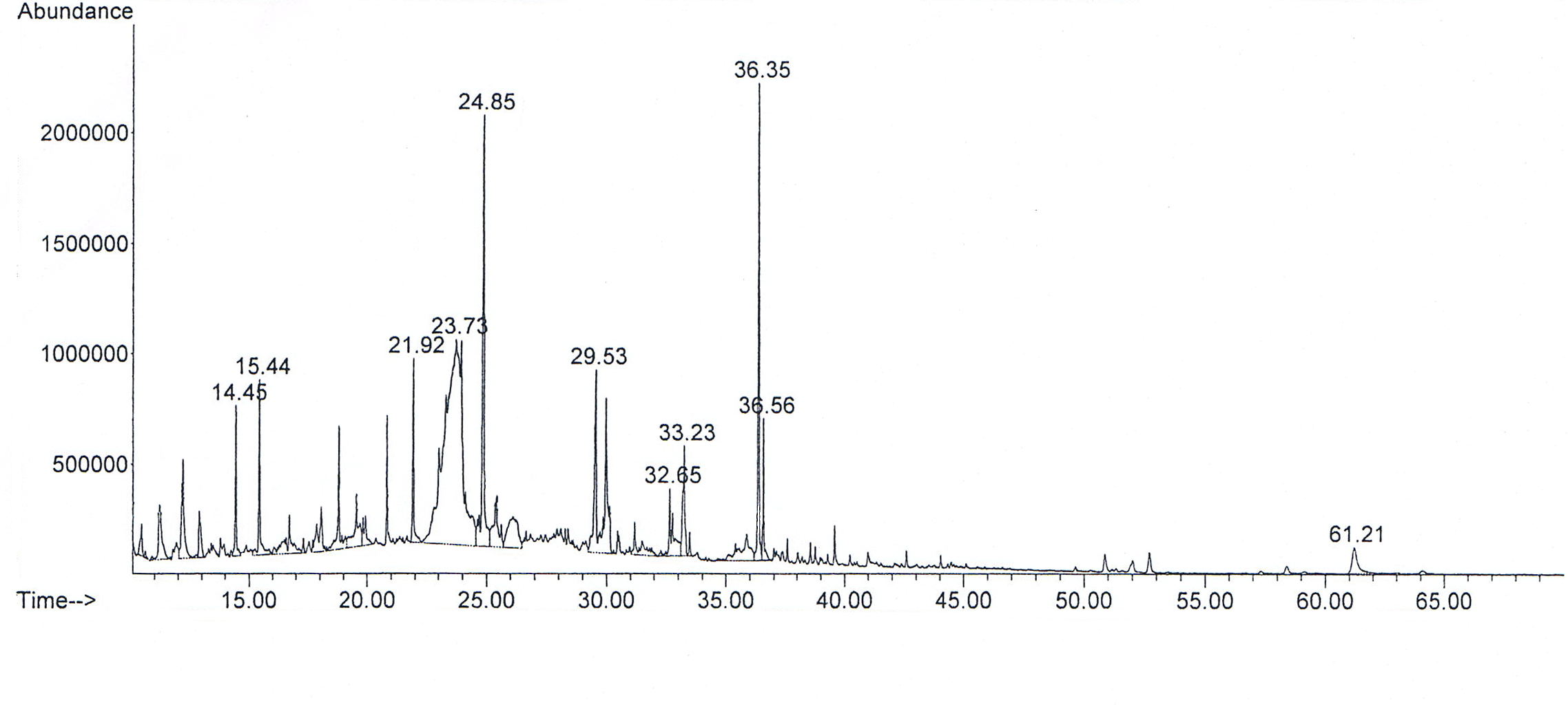


(B)


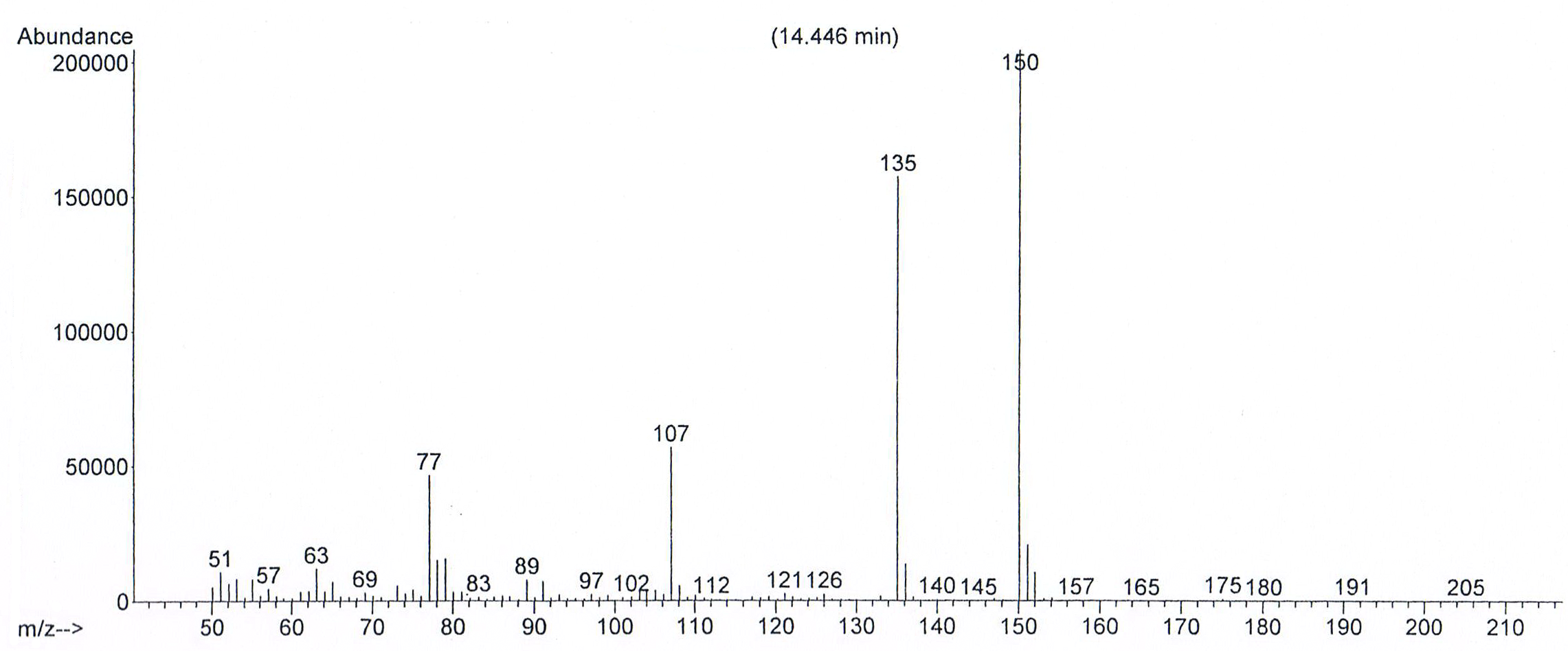


(C)


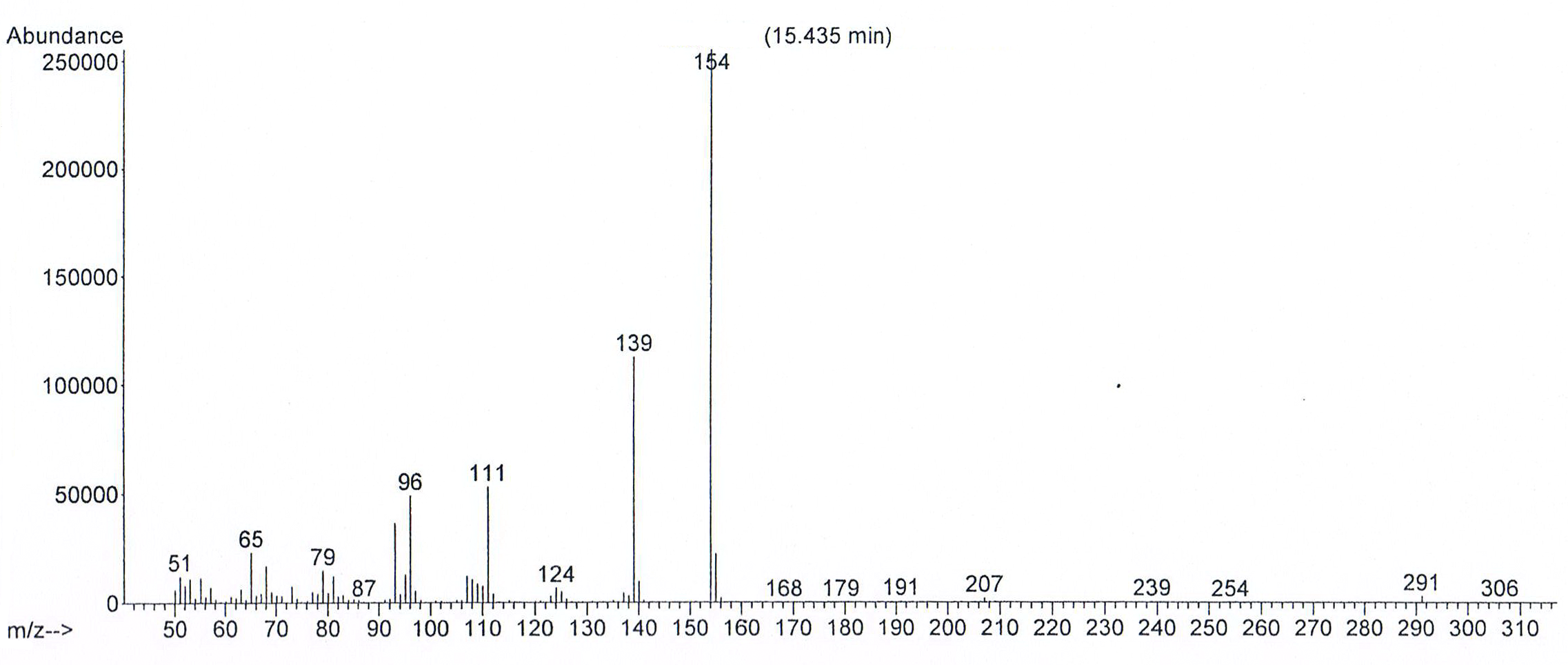


(D)


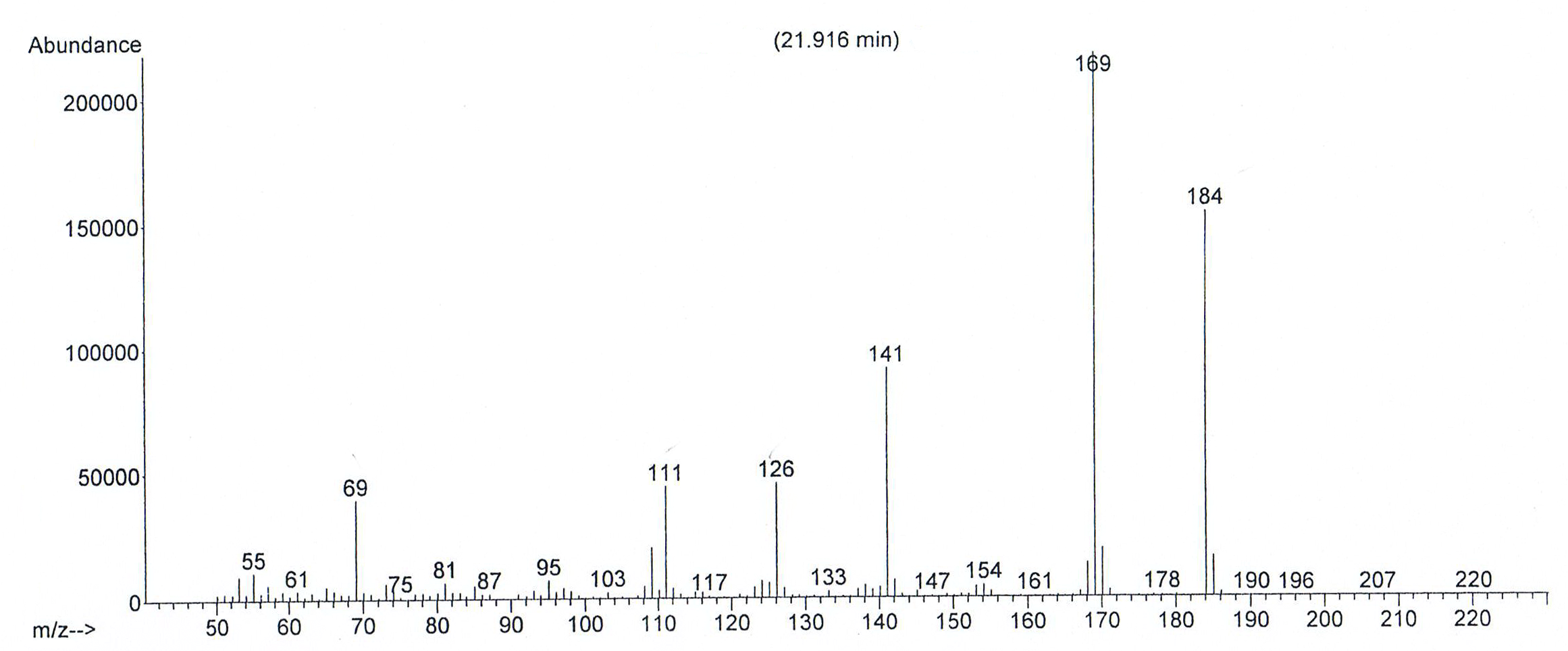


(E)


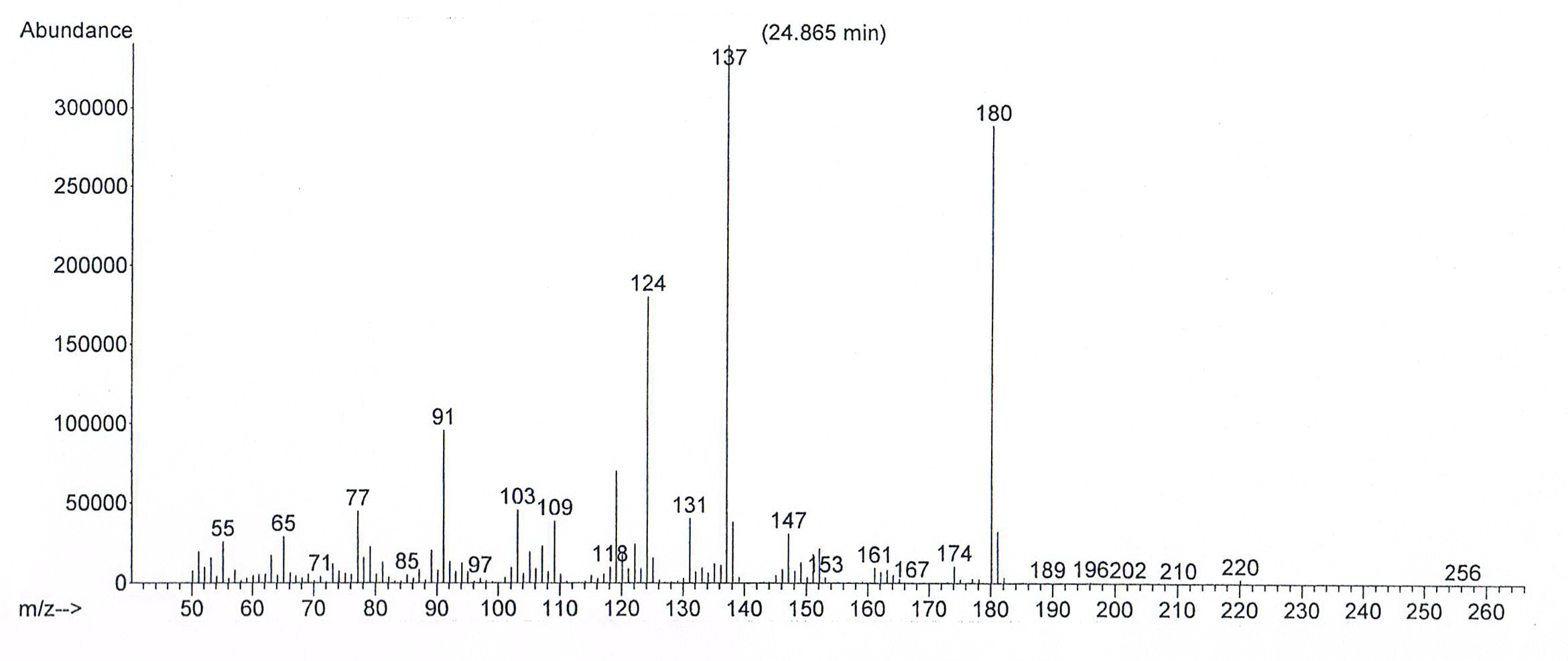


(F)


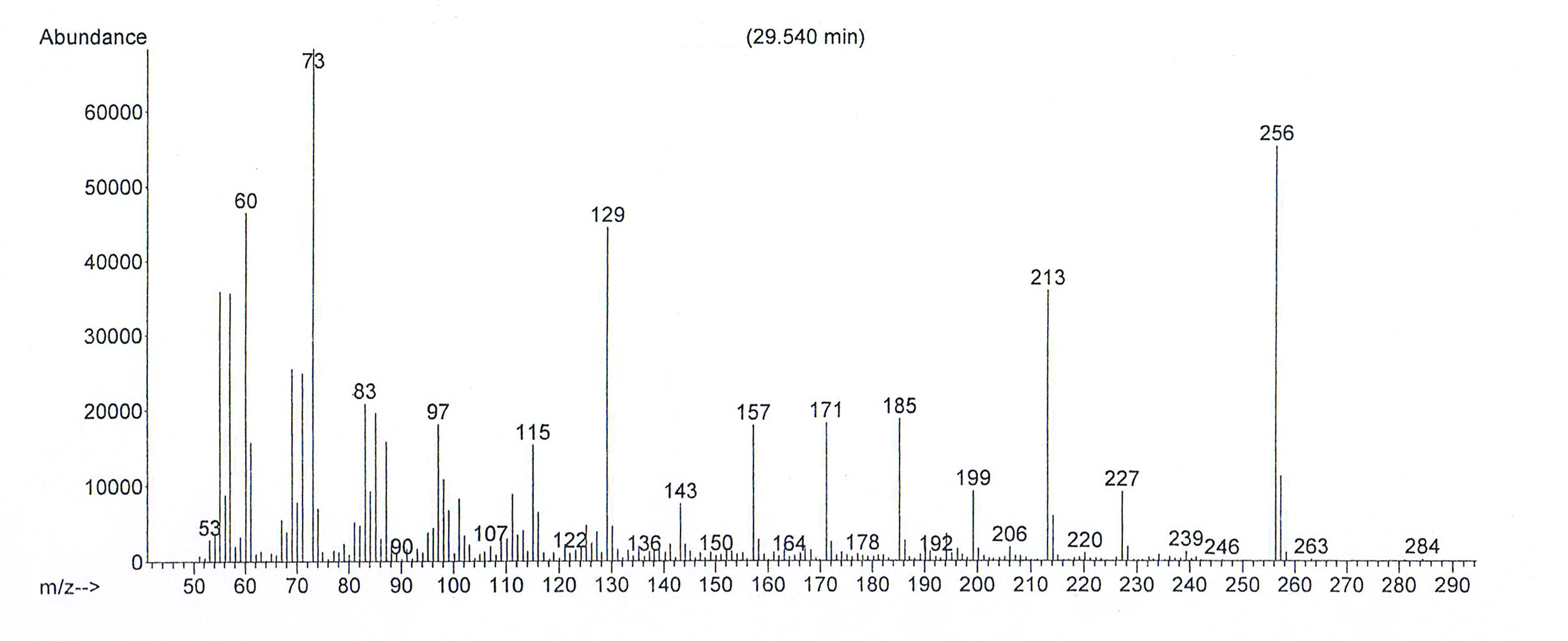


(G)


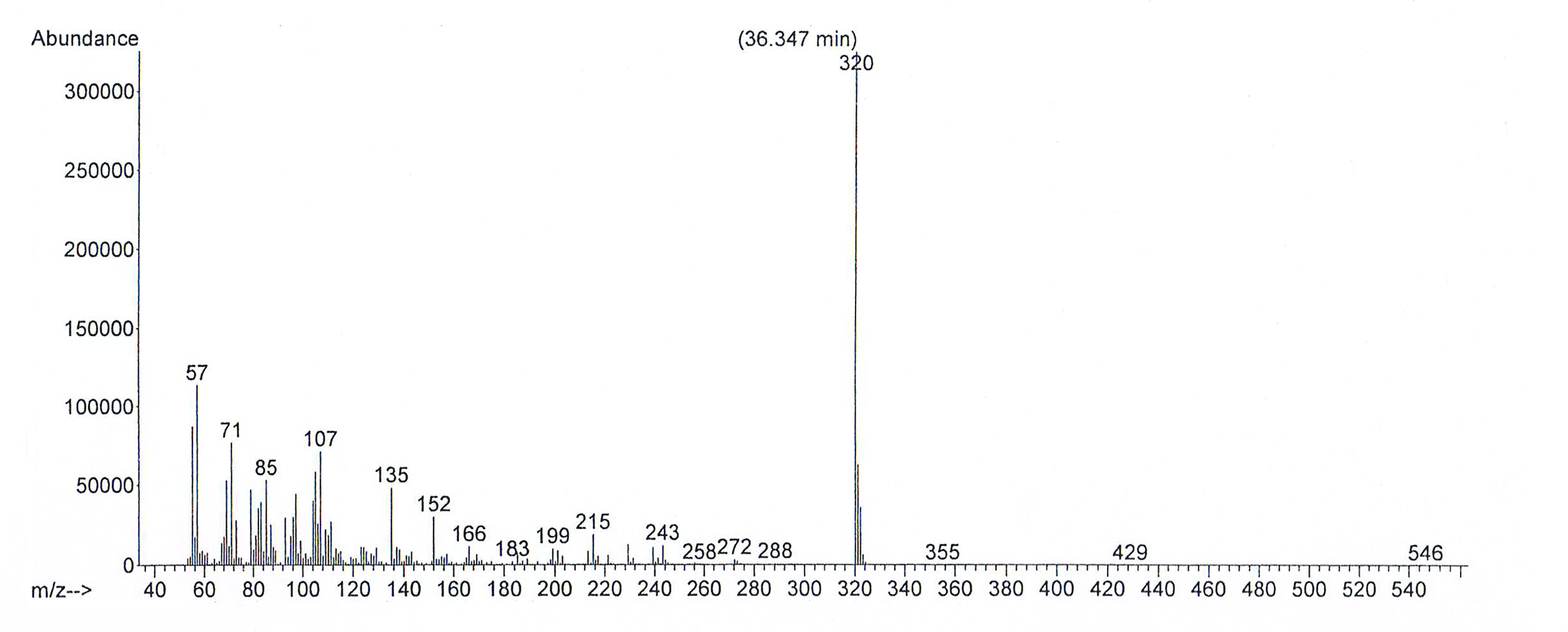


**Additional file 2**: (A) GC-MS chromatogram of 10 mg/mL *D. winitii* crude extract in DMSO. (B-G) Mass spectra of the crude extract with a respective retention time of 14.45, 15.44, 21.92, 24.85, 29.53 and 36.35 min.
