# Supplementary material for: Evaluation of the anticancer potential of six herbs against a hepatoma cell line
Source: Chin Med. 2012 Jun 10;7:15. doi: 10.1186/1749-8546-7-15 (PMC3502167; doi:10.1186/1749-8546-7-15)
Supplement: Additional file 3 — (A) GC-MS chromatogram of 10 mg/mL A. harmandii crude extract in DMSO. (B-F) Mass spectra of the crude extract with a respective retention time of 14.43, 24.88, 29.6, 39.10 and 42.17 min. [file 1749-8546-7-15-S3.doc]

(A)


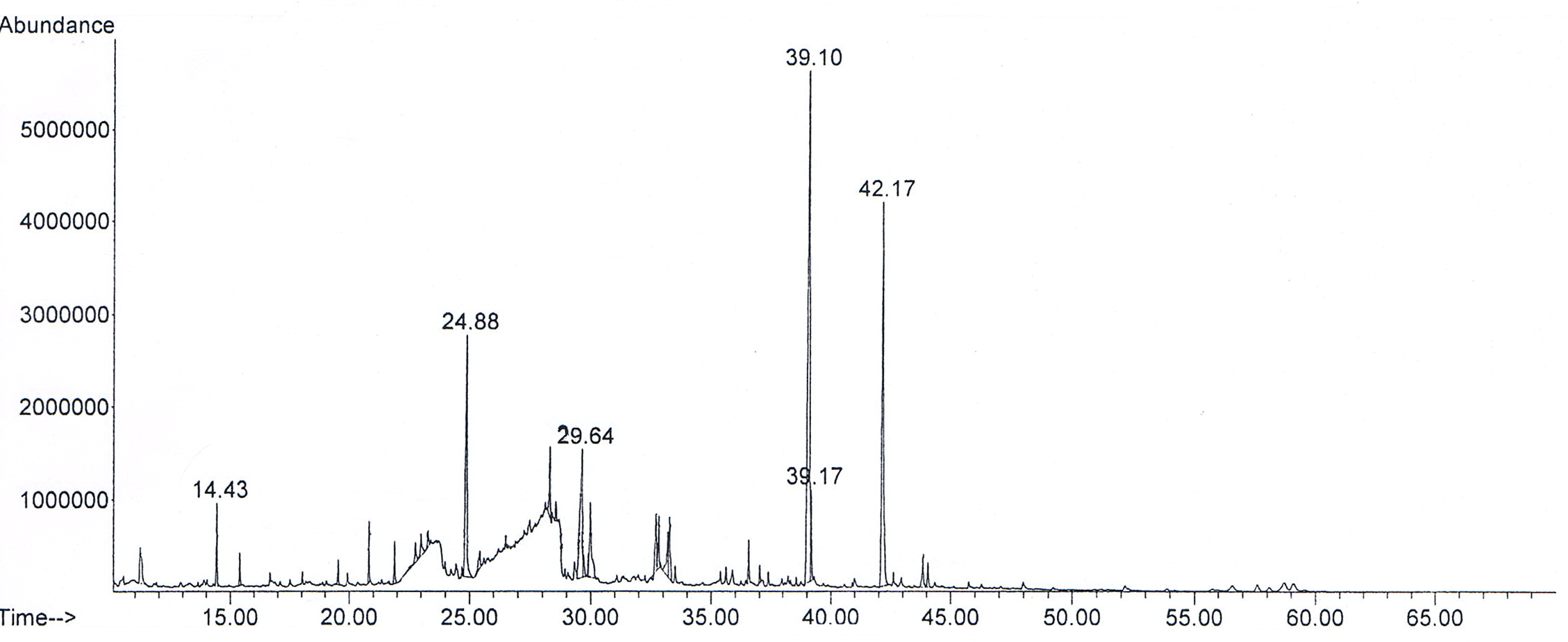


(B)


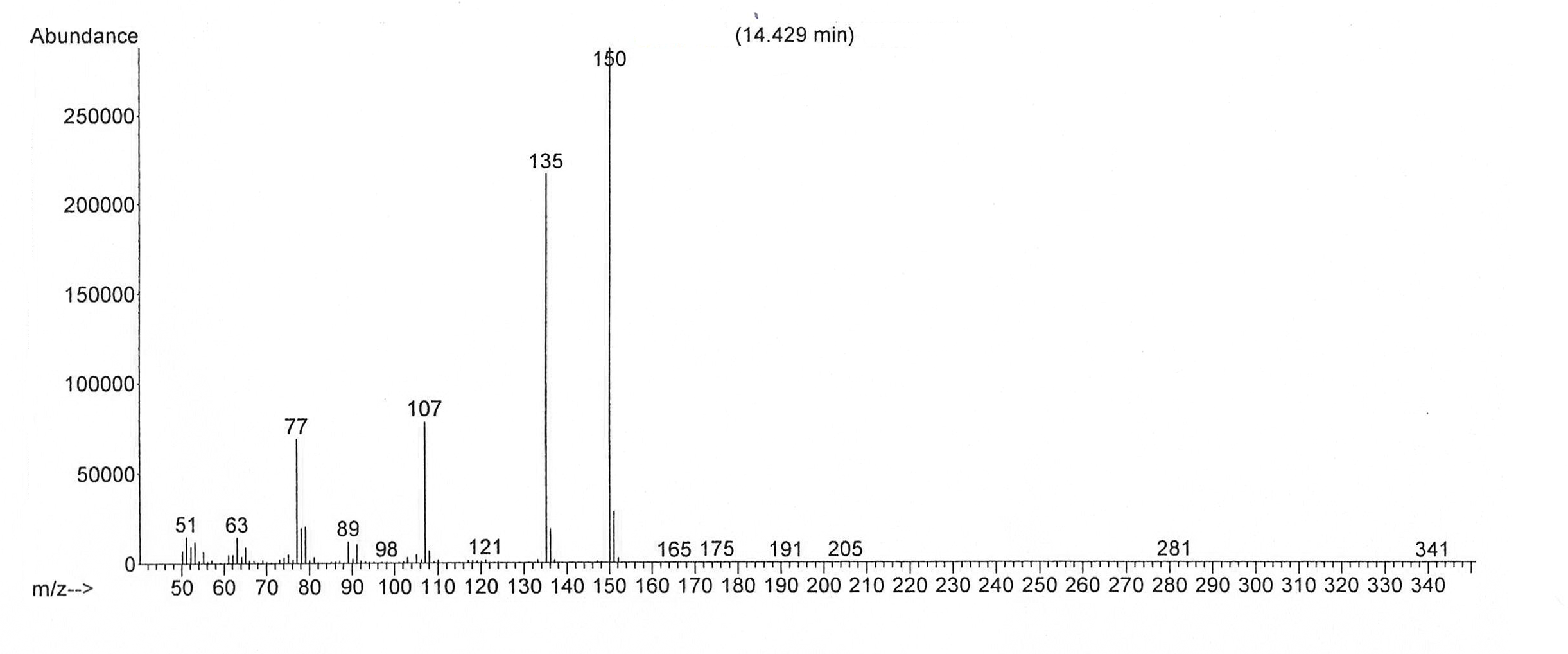


(C)


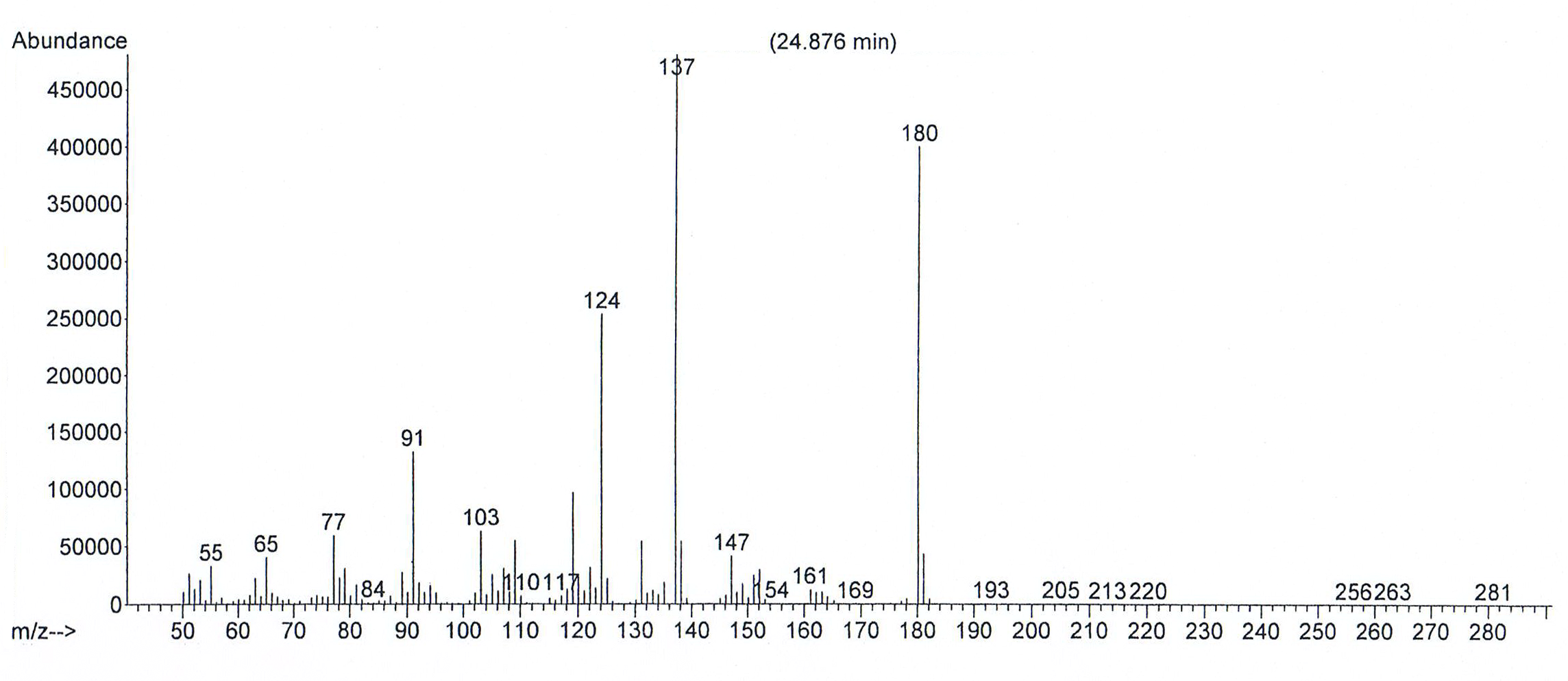


(D)


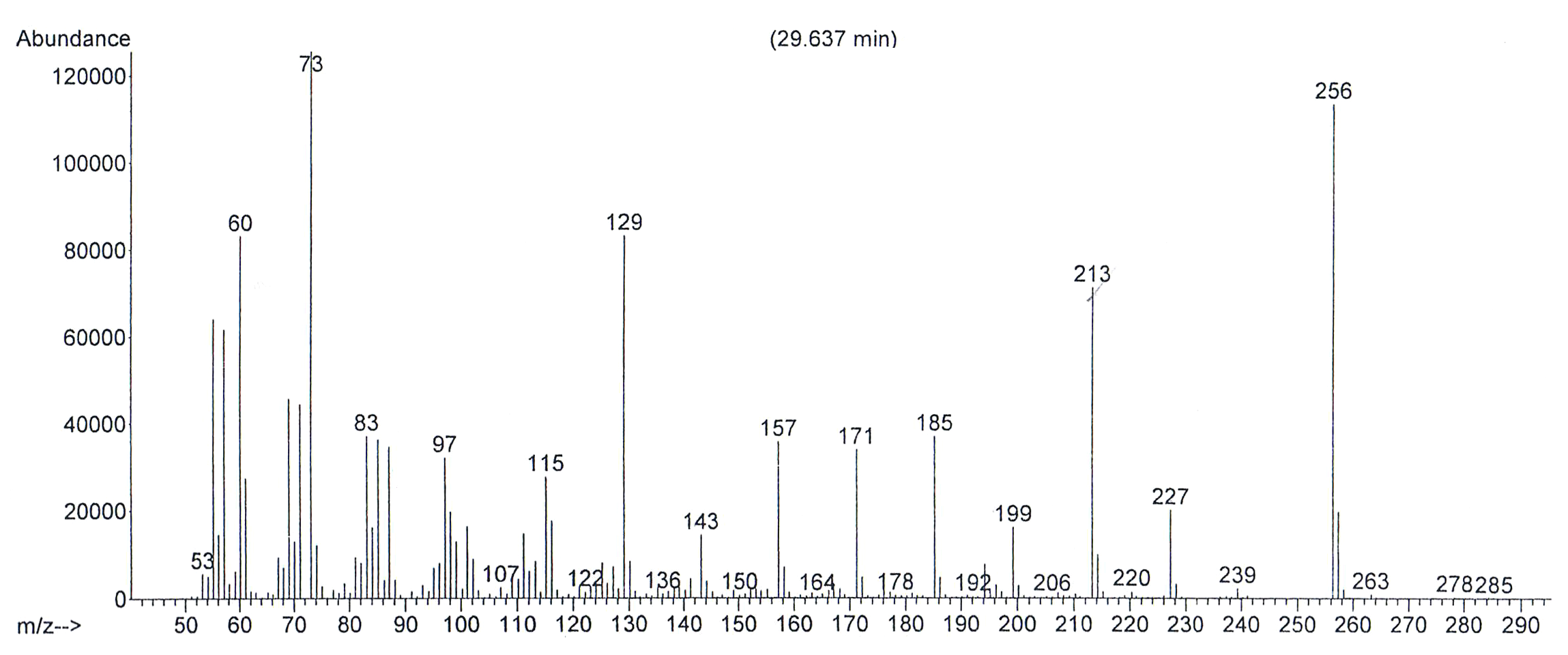


(E)


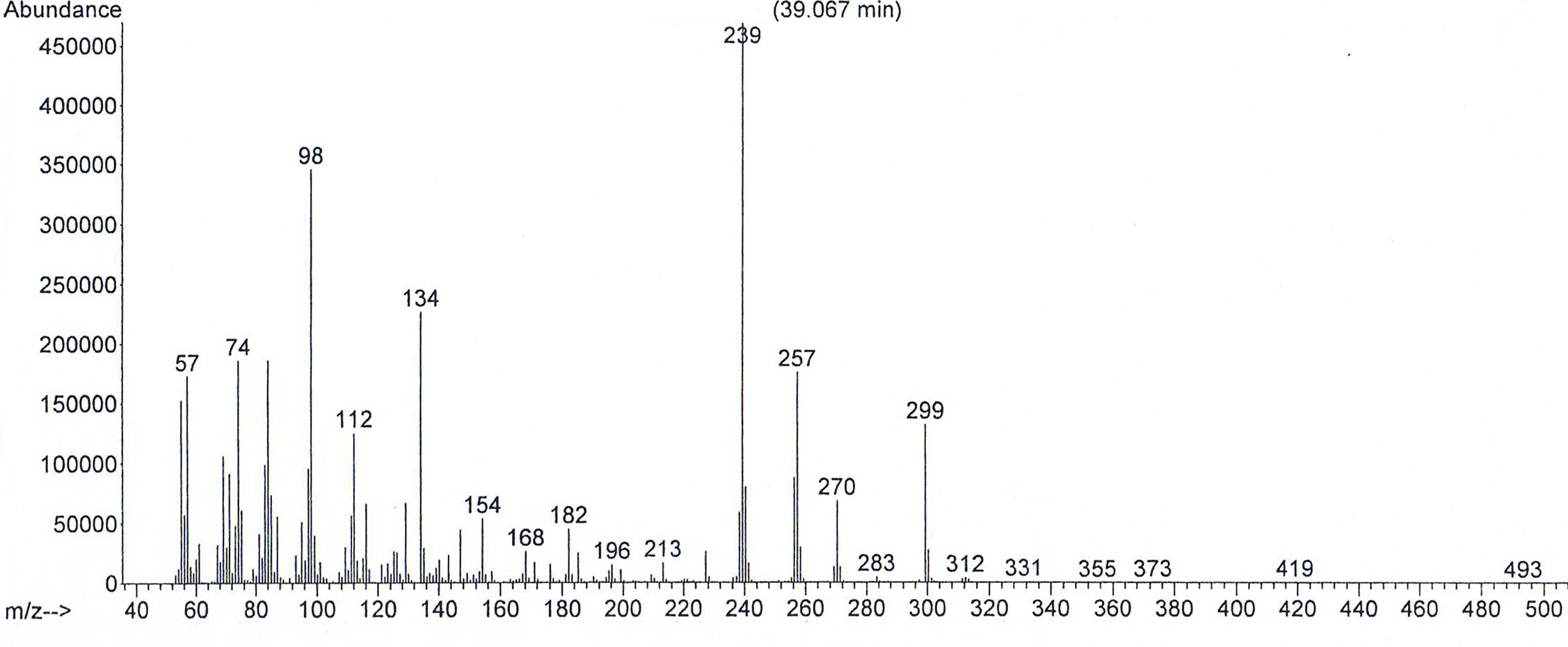


(F)


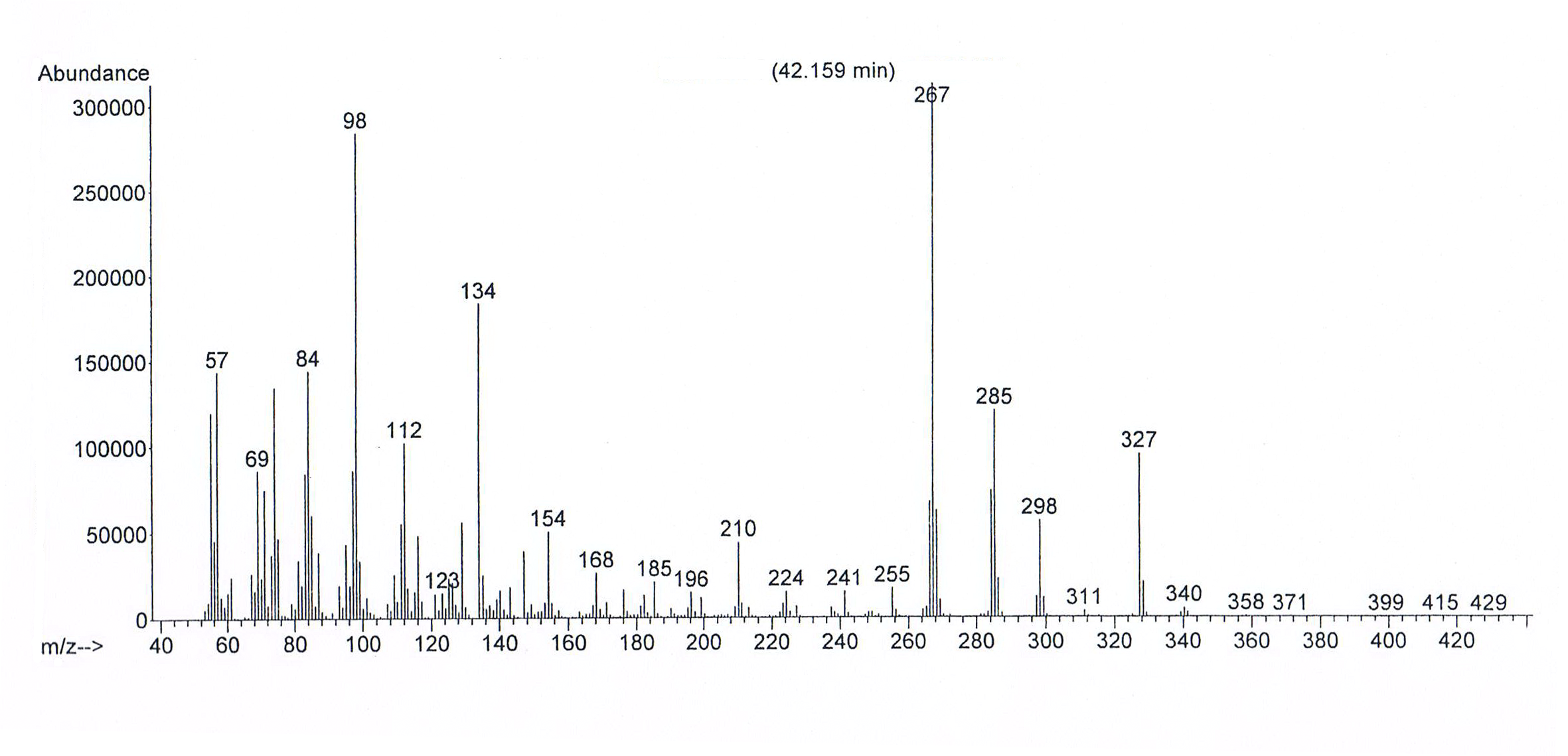


**Additional file 3**: (A) GC-MS chromatogram of 10 mg/mL *A. harmandii* crude extract in DMSO. (B-F) Mass spectra of the crude extract with a respective retention time of 14.43, 24.88, 29.6, 39.10 and 42.17 min.
